# Development and evaluation of an in vivo dose‐based monitoring system for electron FLASH radiation therapy

**DOI:** 10.1002/mp.70549

**Published:** 2026-06-30

**Authors:** Justin DeFrancisco, Matthew Richeson, Tomaj Javidtash, Chris Bartee, Tianjun Ma, Siyong Kim

**Affiliations:** ^1^ Department of Radiation Oncology School of Medicine Virginia Commonwealth University Health System Richmond Virginia USA; ^2^ Medical Physics Department Virginia Commonwealth University Richmond Virginia USA

**Keywords:** Flash, in vivo, scintillator

## Abstract

**Background:**

FLASH radiotherapy requires further preclinical and clinical investigation to establish its biological effectiveness and define optimal beam parameters. In conventional (CONV) radiotherapy, redundant beam termination systems are a cornerstone ensuring patient safety, yet analogous safeguards for FLASH delivery are not well established, creating a critical barrier to safely enabling such studies.

**Purpose:**

To develop and evaluate a real‐time, in vivo, point‐dose monitoring system capable of terminating electron FLASH beam delivery as an additional monitoring system on a modified medical linear accelerator (LINAC).

**Methods:**

A decommissioned LINAC was modified to deliver electron FLASH beams with stable dose per pulse (DPP) at 300 Hz pulse frequency. A commercial plastic scintillation detector system was adapted through hardware and firmware modifications to enable pulse‐based and dose‐based beam termination via the LINAC MLC interface. The detector was cross‐calibrated against radiochromic film under FLASH conditions. System performance was evaluated through measurements of control accuracy, and detector response as a function of DPP.

**Results:**

Stable electron FLASH delivery was achieved with an average dose rate of 127.5±14.91 Gy/s and an approximate beam energy of 5.2 MeV. Pulse‐based control terminated delivery within +3 pulses of the requested value (requested 1‐20 pulses), with overshoot attributable to downstream circuitry latency. Dose‐based control agreed with film measurements within 1.11±0.81 Gy for surface‐based control (in vivo setup) and −1.45±0.38 Gy at depth (stable dosimetry) (tested dose deliveries between 2‐15 Gy). The detector response versus DPP in the FLASH range (0.11–0.78 Gy/p) could be roughly approximated as linear before detector saturation, with only marginal improvement seen when using quadratic fitting.

**Conclusion:**

A modified scintillation‐based system was implemented as a real‐time in vivo beam termination mechanism for electron FLASH radiotherapy under stable DPP and specific experimental conditions. While not intended for primary beam control, the system may provide a practical redundant safety layer for mitigating gross delivery errors in experimental and translational FLASH applications.

## 1 INTRODUCTION

FLASH radiation therapy is an emerging modality for cancer treatment that involves delivering an ultrahigh dose rate (UHDR) at a minimum of 40 Gy/s, compared with approximately 0.03 Gy/s used in conventional (CONV) radiotherapy.^1^ The principal motivation for this substantial increase in dose rate is to induce the so‐called “FLASH effect,” in which normal tissue complication probability (NTCP) is reduced while tumor control probability (TCP) is purportedly preserved. Evidence of this counterintuitive dose‐rate effect was first reported in the 1960s.^2^ and subsequently in the 1970s.^3^ However, technical inability in achieving the required high dose rates to induce the “FLASH effect” hindered further examinations, until reintroduced by Favaudon et al. in 2014.^4^ Numerous biologically based published studies support the existence of normal tissue sparing at UHDRs,^5^, ^6^, ^7^ while others are speculative.^8^, ^9^, ^10^ The recent abundance of research in this field has sparked the publication of several biologically focused review articles,^4^, ^11^, ^12^ as well as FLASH dosimetry review articles.^13^, ^14^, ^15^, ^16^ Aside from possible normal tissue sparing, FLASH radiotherapy offers many other benefits. Reduced NTCP may potentially permit dose escalation.^5^ Additionally, increased dose rate, hence extremely short delivery times, will allow for mitigation of the effects of patient motion and reduce treatment duration, thereby improving patient comfort, delivery uncertainty due to motion, and clinical throughput.^5^ The underlying mechanism of FLASH remains uncertain, and substantial preclinical and translational works are required to enable clinical translation.

Achieving this translation requires not only reliable beam generation and overcoming challenging dosimetry,^16^ but also robust and fail‐safe beam control. In CONV radiotherapy, redundancy is a fundamental principle, enforced by law and international bodies via independent monitoring and termination systems such as dual transmission ion chambers, backup timers, and emergency stop buttons.^17^, ^18^, ^19^ These systems ensure that failure of a single control pathway does not result in clinically significant overdose. However, ion chambers are incompatible with UHDRs due to saturation effects with high DPP requiring unconfirmed corrections.^20^, ^21^, ^22^

In FLASH radiotherapy, the applicability of conventional safety strategies is fundamentally limited. The extremely short delivery times on the order of milliseconds render human intervention mechanisms such as emergency stop buttons ineffective. Even under optimal conditions, the human reaction time of ∼300 ms^23^ would correspond to 12 Gy overdose at minimum FLASH dose rates (40 Gy/s), representing a clinically unacceptable overdose. Additionally, interrupting FLASH delivery mid‐treatment may compromise its biological benefit.^24^ While backup timer clock speeds have potentially sufficient temporal resolution, Konradsson et al.^24^ tested a three‐tiered control strategy consisting of a monitor unit chamber, a pulse counter, and the LINAC's built‐in timer; they concluded that reliance on the timer as a tertiary control will result in unacceptable overdose. A preset time does not deterministically map to the delivered dose in FLASH, since the dose depends more heavily on the pulse repetition frequency (PRF), syncing of the clock with the LINAC pulses, and the dose per pulse (DPP) (and by extension the pulse height and pulse width). In addition, most LINAC timers only accept a value down to a minimum. In the LINAC used in this study, the minimum time allowed is 0.6 s, incompatible with FLASH delivery times. The lack of redundancy is particularly pronounced in modified‐LINAC electron FLASH systems proposed by Rahman et al.^25^, where numerous interlocks need to be overridden to enable beam delivery, resulting in operation beyond originally intended machine parameters^25^, ^26^ ^.^ The current study utilizes one of these systems.

Recent efforts in FLASH beam control have focused primarily on the development of primary monitoring systems, including beam current transformers^27^ and ultrathin transmission ion chambers.^28^ While these approaches are essential, redundancy remains underdeveloped. Importantly, point‐based detectors such as scintillation systems are not well suited for primary beam control due to their limited spatial sampling. However, their compact form, real‐time response, no bias voltage, and broad research works,^29^, ^30^, ^31^ suggest that they are well suited for use as an independent, terminal, in vivo safety monitor to prevent gross delivery error from occurring due to primary system failure.

Accordingly, the purpose of this work is to develop and evaluate a real‐time point‐dose in vivo monitoring system capable of independently (of primary control circuitry) terminating beam delivery in a modified LINAC electron FLASH system. This system is based on minimally modifying a commercially available plastic scintillation detector system (Blue Physics LLC, Tampa, Florida), enabling both pulse‐based and dose‐based beam termination through an external hardware interface and existing LINAC signals independent of the primary monitor chambers termination pathway. By slightly modifying an existing system, the result will be more reproducible in other studies and increase faster translational ability. Blue Physics was chosen due to its temporal resolution of its internal microcontroller unit (MCU), with a clock speed of 120 MHz. The acquisition unit integrates charge on a 1.8 nF capacitor over a programmable window of 700 μ s, and resetting in 10 μ s. After acquisition, the acquired data is displayed in real‐time on an associated laptop for analysis, which can also be used to easily alter the MCU code. The objective of design is not to replace primary control systems, but to provide an additional safety layer capable of mitigating gross delivery errors during experimental and translational FLASH studies.

To support this application, the detector system was characterized under FLASH conditions, including crosscalibration to radiochromic film and evaluation of its response as a function of DPP. The performance of the system as a beam termination mechanism was assessed through controlled delivery experiments. Together, these results establish the feasibility of using a modified scintillation detector as an independent, in vivo safety device for electron FLASH radiotherapy.

This article includes a fair number of terms either emerging or infrequently used thus, a glossary is provided for convenience (Table 1).

**TABLE 1:** Glossary.

Terminology (Abrv.)	Definition
Automatic frequency control (AFC)	A feedback loop radiofrequency (RF) control system of medical LINACs, adjusting RF to compensate for thermal expansion of the waveguide and changes in energy setting to stabilize DPP—that is, the cause of ramp‐up.
Blue physics flash (BPF)	A modified version of the commercial Blue Physics scintillation detection system to enable accurate FLASH readings and pulse/dose‐based control of a FLASH beam—Mainly altering capacitors, MCU code, and adding extra external circuitry to enable interfacing with the LINAC.
Conventional (CONV)	Radiation delivery average dose rate ∼0.03 Gy/s.
Dose per pulse (DPP)	The dose in an individual bunch of radiation accelerated and delivered, applicable to all pulse‐based medical LINAC systems—Delivery is not continuous.
Dose‐rate (DR)	Dose rate of radiation delivery (typically in Gy/s or cGy/s).
FLASH	Radiation delivery average dose rate > 40 Gy/s.
Microcontroller unit (MCU)	A miniature computer with specific firmware on a signal chip with CPU, memory, and programmable input‐output to control systems.
MLCHOLDOFF	MLCHOLDOFF: A specific signal on a Varian LINAC MLC interface board that can assert a beam hold signal by placing the triode gun grid current out of phase with the waveguide RF, designed for step‐and‐shoot IMRT to prevent delivery while MLC leaves shift to the next treatment position.
Pulse repetition frequency (PRF)	The frequency in which bunches of radiation are delivered. In this study, 300 Hz is used.
Ramp‐up	The initial pulses (∼ 4–6) of medical LINACs consist of bunches where the DPP is lower until stabilized by the AFC.

## 2 METHODS

### 2.1 FLASH Beamline System

At our institution, Virigina Commonwealth University (VCU), a decommissioned Varian C‐Series 21EX LINAC (Palo Alto, California) was modified to enable electron FLASH delivery at the isocenter. As mentioned, the modification procedure was established by Rahman et al.^25^ Briefly, the LINAC is operated in photon mode while removing attenuating beamline components (target, scattering foil, and port cover), allowing direct delivery of the high‐current electron beam.

With these modifications, a ramp‐up period still occurs.^25^ These ramp up pulses will not consist of dose rates in the FLASH regime and need to be removed. To achieve stable FLASH delivery, the automatic frequency control (AFC) system was disabled, and a fixed RF frequency was manually selected. This approach produces pulses with approximately constant DPP, consistent with the intended operating regime of FLASH delivery. Independently, several beam interlocks were overridden at the console. All measurements, unless otherwise specified, were performed using a 10 × 10 cm^2^ applicator and insert at 100 cm SSD with a nominal PRF of 300 Hz, pulse width of 5 µs and pulse height (gun current amplitude) of 1.7 V. Due to pulse dropping behavior in this LINAC model, the instantaneous PRF was closer to 360 Hz for most pulses.

### 2.2 Evaluation of FLASH beam spatial characteristics

The spatial characteristics of the electron beam were evaluated using radiochromic film. Percent depth dose (PDD) curves were measured by sandwiching EBT3 (Ashland, Wilmington, Delaware) film between solid water slabs aligned with the beam axis. Lateral dose profiles were obtained by placing film perpendicular to the beam at 1.2 cm depth with appropriate backscatter.

Films were scanned then analyzed using ImageJ, with profiles extracted and subsequently smoothed to reduce rasterization effects from the scanner. All plots were normalized according to standard conventions.

### 2.3 Development of the Blue Physics FLASH (BPF) control system

A commercial plastic scintillation detector system (Blue Physics LLC, Model 11) was modified to enable real‐time beam termination. The system consists of two independent optical channels: a scintillation channel coupled to optical fiber and a reference optical fiber channel for Cherenkov correction. The scintillation material included is a Bicron Fiber model 10 (BCF‐10) scintillator with a polystyrene‐based core and thermoplastic (PMMA) cladding. BCF‐10 has a 432 nm emission peak and 2.7 ns primary decay time. To interface the detector with the LINAC, a hardware pathway was developed linking the detector's included digital output to the multi‐leaf collimator (MLC) interface board. The 3.3 V direct current output signal was amplified to 5 V and passed through an optoisolator before connection to MLC interface test points (TP1, TP2, W3). Assertion of this signal activates the MLCHOLDOFF input, which modulates the triode gun grid timing relative to the RF waveform, disturbing coincidence and preventing electron injection into the waveguide while maintaining RF loading. MLCHOLDOFF is applied in step‐and‐shoot IMRT techniques, to pause the treatment until the MLC leaves get to the next position. In the developed control system, an operator must first start the measurement with Blue Physics, then manually fire the beam from the control console. Once the beam is on, the acquisition unit's signal will assert the beam hold state once a desired measurement has been recorded. In this state, the waveguide is still being loaded with RF and the gun is pulsing, so the LINAC operator must manually fully shut off the beam. However, after MLCHOLDOFF is asserted by the scintillator, no radiation exits the LINAC. Finally, the measurement must be manually stopped on the Blue Physics acquisition unit, as the device keeps measuring even after shutting off the LINAC. This feature is used to verify the accuracy of control, along with film dosimetry.

The detector firmware was modified to perform real‐time pulse counting and dose calculation. Signal acquisition is based on charge integration using a capacitor readout system. Voltage readings above a defined threshold (−0.05 V) were identified as radiation pulses. The Blue Physics system intentionally applies a reverse bias to the capacitor to slightly increase the dynamic range of the 12 V capacitor to 12.2 V. Individual pulses of the LINAC are detected as much higher voltage readings, in most of this study on the order of 5 V. Dose was calculated from measured charge using:

(1) $$Q=C\cdot \left[\left(V_{1}+0.2\right)\;-\;\left(\left(V_{2}+0.2\right)\;\cdot \;ACR\right)\right]$$

(2) D=kQ

where *Q* is the charge in nanocoulombs, $V_{1}$ and $V_{2}$ are the voltages from channel 1 (scintillator and fiber) and channel 2 (fiber only, for Cherenkov correction), respectively, *ACR* is the adjacent channel ratio correction factor discussed in section 2.5, and *k* is a cross‐calibration factor against film in FLASH. The 0.2 factor “zeros” out the capacitor reading to be positive over the whole 12.2 V dynamic range. These equations are input into the MCU and calculated in real‐time, each time a signal above the threshold is collected. Once the user‐defined dose and/or pulses are achieved, the MCU will send the output signal to the external hardware, and subsequently the LINAC for beam off assertion. The graphical user interface (GUI) was revised to include user‐inputs for desired dose, desired pulses, threshold value, and calibration factor.

Since the voltage sampling of Blue Physics is not in phase or the same frequency as the LINAC delivery, sometimes the acquisition unit samples the capacitor simultaneously as a pulse is being delivered. When this occurs, a backup capacitor is employed to ensure full collection of the charge, then the charge is transferred back into the readout capacitor to be included in the next sample. In principle, with the backup capacitor method, no charge is lost and dose control can be made accurate. During FLASH operation, the original 1.8 nF backup capacitor exhibited voltage clipping due to high instantaneous charge. This component was replaced with a 47 nF capacitor, eliminating saturation and ensuring complete charge capture during capacitor reset cycles. Additionally, the dual‐capacitor system results in an error in simple pulse counting, as a single pulse can be double counted by separation of a single pulse into adjacent samples. Pulse counting logic was refined to prevent double counting by requiring a threshold crossing preceded by a subthreshold sample.

The findings presented in this work are from measurements after all modifications to the Blue Physics system were completed. For preliminary data before all the modifications took place, interested readers should refer to a cited thesis.^32^

### 2.4 Calibration of GafChromic films

**FIGURE A1:**
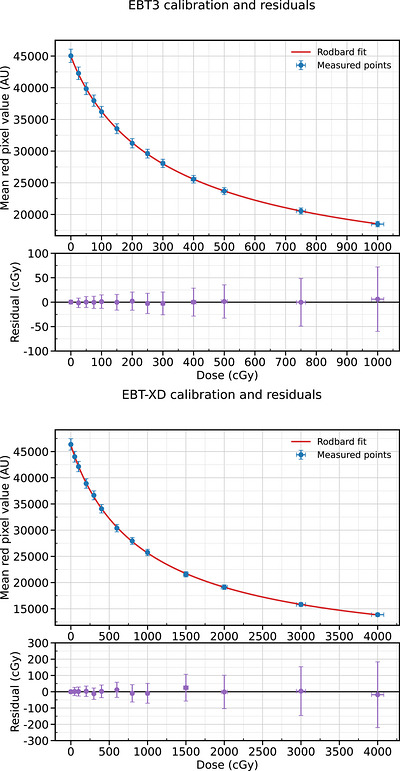
Calibration curves relating mean red pixel value to dose of EBT3 (left) and EBT‐XD (right) films fit with the Rodbard function.

To calibrate the sheets of film for FLASH use, they were irradiated with CONV 6 MeV electrons on a Varian TrueBeam. EBT3 and EBT‐XD (Ashland, Wilmington, Delaware) films were calibrated in the 0–10 and 0–40 Gy range, respectively. For CONV irradiation, the reference condition was utilized consisting of a 10 × 10 cm^2^ applicator/insert, 100 cm source to surface distance (SSD), 1.2 cm depth in solid water with backscatter. In these conditions, the number of monitor units entered in the LINAC console can be directly related to the dose delivered to films in cGy. After irradiation, a 24‐hour wait period was conducted as recommended.^33^ After 24 h, films were scanned using an Epson Perfection V800 Photo scanner in transmission mode with 48 bit 3‐color channel (red‐green‐blue), 75 dpi, and all color enhancement settings turned off. For analysis, ImageJ software was used to isolate the red channel as recommended due to improved sensitivity.^34^, ^35^, ^36^ Regions of interest (ROI) of similar size (about 1 × 1 cm^2^) were made on scanned films to extract a mean red pixel value. Calibration was conducted by fitting a function to the pixel values versus their respective doses taken from monitor units. The Rodbard function was chosen for fitting due to its invertibility for direct use to easily convert future pixel value measurements to dose (See Appendix A and Figure A1).^34^, ^35^

### 2.5 Cross calibration of scintillator in FLASH

To convert BPF samples of capacitor voltage to dose using Equation 1 and 2 above, the ACR correction factor and cross calibration to film is needed. ACR accounts for the differences in Cherenkov sensitivity between the two optic fiber channels. BPF simply subtracts the optic fiber reading from the scintillation plus optic fiber reading to remove Cherenkov from the primary channel, under the assumption that the two channels produce the exact same magnitude of Cherenkov. The quantification of this correction factor is an active area of research. In CONV, the Blue Physics detector has recently been published illustrating several different measurement methods to determine ACR, all resulting in similar values.^37^ The method chosen in this work was to irradiate the detector twice on a Varian TrueBeam delivering 6 MV photons with a 3 × 6 cm^2^ field. Individual jaws had the following settings, where the detector tip was placed on the crosshairs: *X*1 = 1.5 cm, *X*2 = 1.5 cm, *Y*1 = 1.5 cm, *Y*2 = 4.5 cm. The elongated jaw setting is initially placed in the direction of the optic fiber for the first reading (0‐degree collimator rotation), until subsequently being rotated 90 degrees for a second reading. This irradiation scheme is expected to deliver the same dose to the scintillation material, while irradiating different lengths of the transport fibers. From these two irradiations, ACR is calculated via Equation 3:

(3) $$ACR=\left(V_{1}^{0^{\circ }}-V_{1}^{90^{\circ }}\right)/\left(V_{2}^{0^{\circ }}-V_{2}^{90^{\circ }}\right),$$

where $V_{1}^{0^{\circ }}$ is the voltage from channel 1 (scintillation coupled to optical fiber) for the 0 degree collimator rotation, $V_{1}^{90^{\circ }}$ for the 90 degree rotation, and $V_{2}^{0^{\circ }}\&\;V_{2}^{90^{\circ }}$ for the channel 2 reading of just the optical fiber for Cherenkov correction for the 0 degree and 90 degree readings, respectively. Importantly, it was assumed in this work that the ACR determined from a CONV 6 MV photon beam applies to a 6 MeV FLASH electron beam since ACR is a property of the detector transport system of visible photons alone.

**FIGURE 1:**
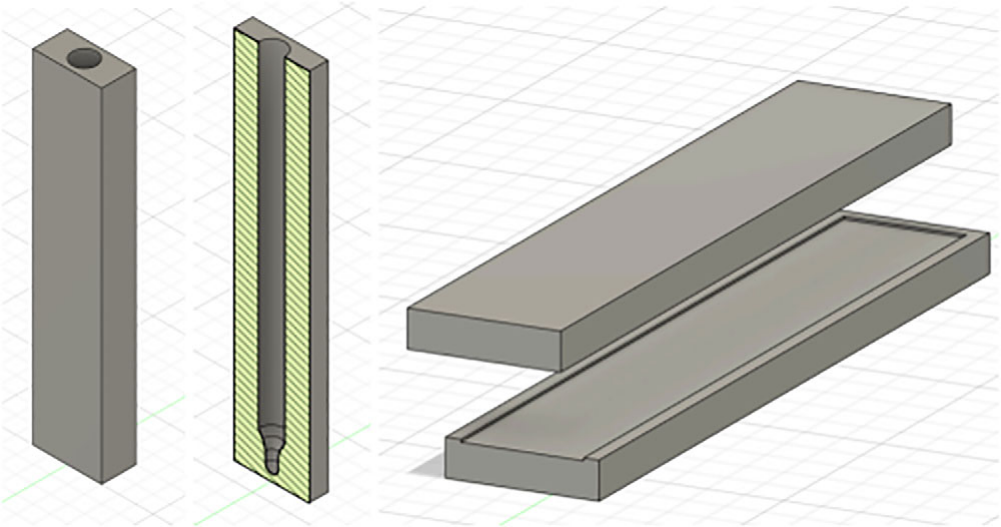
Custom phantoms to house the Blue Physics scintillation detector (left, cross section in the middle) and a strip of film (right) for simultaneous irradiation, designed using Fusion360 CAD software.

Custom phantoms were 3D printed to hold BPF and films equidistant from the central axis to enable simultaneous irradiation. Shown in Figure 1, phantoms were designed using Fusion360 computer aided design (CAD) software. The phantoms are rectangular prisms with a hollow middle created from subtracting the detector models from the prisms at similar depths. For printing setting selection and 3D printing with polylactic acid (PLA), Dremel Digilab software and hardware system were used. A computed tomography (CT) scan of the phantoms was taken, finding a mean Hounsfield unit (HU) of 192.

To calibrate, two different conditions were examined:

1. Detector and film at 1.2 cm depth in water equivalent materials (charged particle equilibrium)
2. Detector at surface with film at 1.2 cm depth in water equivalent materials (in vivo configuration)

Linear relationships between charge and dose with pulsed deliveries between 2 and 40 pulses using pulse‐based control were used to derive calibration factors.

### 2.6 Evaluation of control performance

The system was evaluated for both pulse‐based and dose‐based control. Whichever of the requested values of pulse number or dose entered simultaneously is measured first, the halting signal will be sent. Therefore, the two control systems were tested independently in this work by entering a large requested value for the other control mechanism while testing the other. In this manner, the MCU of BPF is still calculating the number of pulses and dose simultaneously, but stopping the beam based on only the mechanism of interest.

For pulse‐based control, 25 controlled tests were conducted, requesting pulse numbers between 1 and 20. BPF was placed in the custom phantom with 1 cm lateral scatter solid water adjacent, 5 cm solid water backscatter, and an extra 0.7 cm solid water of buildup. The number of pulses detected for control is based on scintillation and optic fiber channel measurements. Therefore, the number of pulses detected can be directly compared between the two channels themselves to evaluate any existence of discrepancy in pulses between them. Additionally, the requested pulses were compared to the actual pulses delivered. Hardware latency was evaluated by requesting the MCU to print the detected pulses at the time of sending the output signal and comparing with the final detected pulses by both independent channels.

Dose‐based control was evaluated in a surface in vivo condition, and at the hypothesized depth of maximum dose for a 6 MeV electron beam in a region of stable dosimetry. The setup for both control tests was identical to those at the time of calibration. Requested doses were 2, 5, 10, and 15 Gy. Each dose point was repeated at least three times in each condition. The measured doses from film were compared to the dose reported by BPF including measurements after the beam was stopped, and the requested doses entered into the BPF system. Results from the multiple trials were averaged for each requested dose, and the relative error determined from the standard deviation in individual trials was combined in quadrature with the uncertainty of the film doses and scintillator doses for reporting.

### 2.7 DPP dependence

To evaluate detector suitability under FLASH conditions, the response of the scintillator was measured as a function of DPP. Single‐pulse irradiations were performed while varying pulse width from 1.7–5 µs and for a few data points to achieve lower or specific DPP values pulse amplitude (gun current) between 1.25–1.7 V. These modulations were performed by adjusting potentiometers in the LINAC circuitry and monitoring the pulse structure with an oscilloscope. DPP was independently measured using a flashDiamond detector (PTW, Freiburg, Germany), and scintillator response was expressed as charge per pulse. The doses per pulse achievable on our system were relatively low (< 1 Gy/p) to accurately measure with film. The flashDiamond detector was chosen due to the ease of use as it can be calibrated by an Accredited Dosimetry Calibration Laboratory (ADCL), and since it has demonstrated linearity with DPP up to at least ∼20 Gy/p.^38^ The ADCL calibration was confirmed via film measurements in FLASH before use. To examine any dependency on pulse size, a custom phantom was printed similarly as previously described to encompass the flashDiamond detector. On top of backscatter solid water, the custom scintillation phantom was placed and surrounded by solid water. The flashDiamond and holder were placed above this, followed by an extra 0.5 cm buildup of solid water. This setup places the flashDiamond and BPF at 0.95 cm (∼depth of dmax) and 1.9 cm depth in water equivalent material (∼60% of max), respectively. Raw flashDiamond readings were corrected to corresponding values at the depth of Blue Physics using the PDD. For this test only, the applicator was removed, and the SSD was set to 79 cm to increase the DPP. Two different jaw settings were examined: 5 × 5 cm^2^ and 7 × 7 cm^2^. Two different field sizes were examined to investigate if the saturation of the detector can be pushed to a higher DPP with less Cherenkov signal generation taking up the allowed 12.2 V of the capacitor.

### 2.8 Uncertainty analysis

Uncertainties were classified as Type A or Type B following the Guide to the Expression of Uncertainty in Measurement (GUM) and are presented in Table 2.^39^ For the flashDiamond detector, the ADCL calibration uncertainty was used, and other detector related contributions, such as signal repeatability, were assumed to be included within the ADCL uncertainty budget. Film uncertainties were based on typical values reported in literature and the specific assumptions described below, rather than a full independent characterization of our workflow.

1. Energy and DPP Dependence: Since films have shown to be DPP and energy independent in the ranges used in this work, no error was considered from these factors.^40^, ^41^
2. Lateral setup error: Lateral setup error was not considered for film readings since users can retrospectively decide where to read out the films with ROIs on the software. For spatial beam characteristic measurements, small setup errors in SSD have been shown to minimally affect the result, so it was ignored.^42^, ^43^
  - For other measurements, the SSD was unchanged after initial setup.
3. Optical density versus time: The change in optical density of film over postirradiation time was not considered, since the darkening rate after 24 hours is minimal, and great care was taken to ensure films were scanned near the 24‐hour mark.
4. Past expiration date use: All films were used within the expiration date range.

**TABLE 2:** Values of uncertainty.

Dosimeter	Quantity	Source	Type (A or B)	Relative uncertainty (*k* = 1)	Determination method
Gafchromic film	Pixel value	Scanner reproducibility	B	0.20%	Ref. 44, 45, 46, 47
		Scanner uniformity	B	2.09%	
		Film uniformity	B	1.20%	Included in manufacturer documents in batches
	Dose	LINAC output for conventional calibration	B	2.00%	Daily QA output variation tolerance
		Rodbard fit	B	1.65%(EBT3), 1.89% (EBT‐XD)	Standard deviation of residuals between known calibration doses and doses predicted from the inverse Rodbard Fit
		Pixel value uncertainty propagated through Rodbard fit	B	Dose‐dependent	[D(PV + σPV)—D(PV—σPV)]/2
Blue Physics Scintillator	Charge	ACR correction (*n* = 3).	A	1.50%	Standard deviation of three repeated ACR measurements
		Lateral setup (2 mm)	B	1.11%	Obtained from Profiles
		Baseline +0.2 V correction	A	0.22%	Standard deviation of repeated baseline measurements
	Dose	Crosscalibration factor, surface	B	2.15%	Relative uncertainty of the weighted‐mean calibration factor, with pointwise uncertainty from film dose and BPF charge uncertainty combined in quadrature
		Crosscalibration factor, depth	B	2.07%	
flashDiamond	Dose	ADCL calibration factor	B	2.50%	From ADCL

## 3 RESULTS

### 3.1 Beam characteristics

**FIGURE 2:**
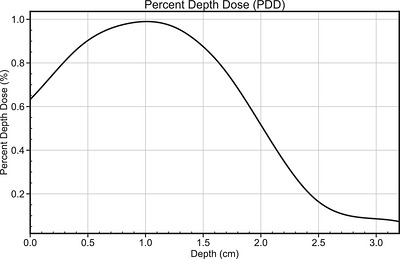
PDD curve of the FLASH beam measured with EBT3 Gafchromic film, smoothed, and normalized to the maximum value.

**FIGURE 3:**
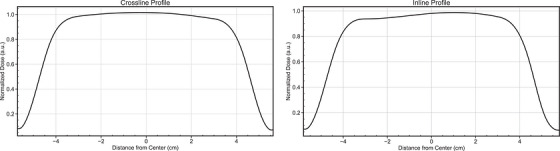
Left: crossline, right: inline profiles of the FLASH beam measured with EBT3 Gafchromic film, smoothed and normalized to the center value.

The modified LINAC successfully delivered stable electron FLASH beams with an average dose rate of 127.5 ±14.91 Gy/s under the reference condition. The measured depth of maximum dose occurred at approximately 1.09 cm, with a practical range of 2.6 cm, corresponding to an effective beam energy of ∼5.2 MeV even though 6 MeV photons were selected at the console (Figure 2). Major components of the typical beamline were altered in this study, many of which could be the cause of the observed discrepancy in energy. Lateral profiles exhibited asymmetry relative to conventional beams, though dose distributions remained sufficiently flat within the central region, even with the absence of the scattering foil (Figure 3). One reason for this could be the presence of the applicator in this study versus other studies where the fields are defined using the jaws. The PDD curve confirmed expected electron beam behavior under modified beamline conditions.

**FIGURE 4:**
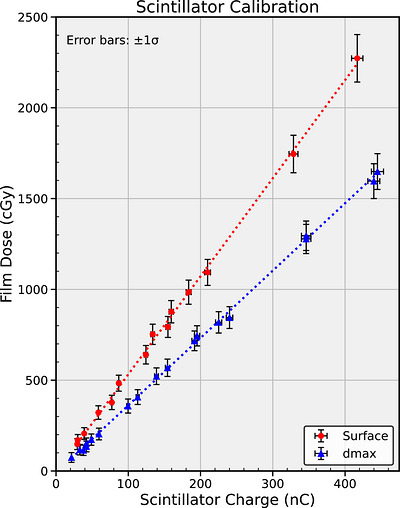
Cross calibration of the BPF in a FLASH electron beam in two separate conditions. Red: Scintillator on the surface, film in 1.2 cm depth of solid water. Blue: Scintillator and film both in depth at 1.2 cm depth in solid water and custom phantoms.

**FIGURE 5:**
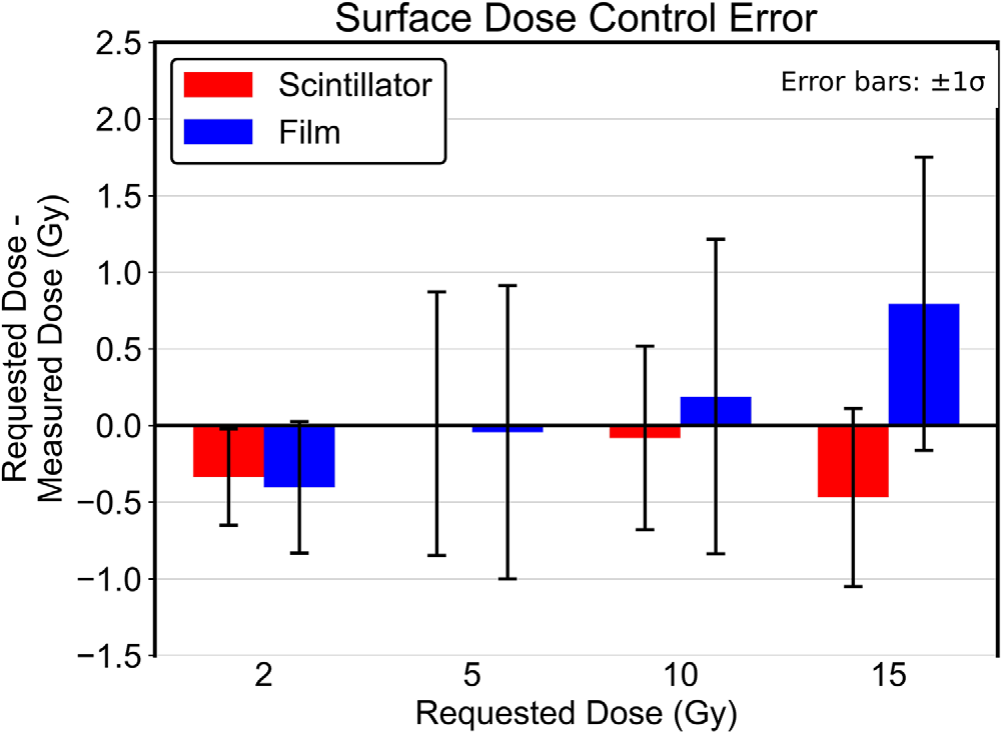
Control error based on deviation to requested dose entered into the BPF detector system for the scintillation detection system reading on the surface and film reading at 1.2 cm depth in solid water. For each requested dose, requested dose—measured dose are displayed as the average values over multiple trials, with the uncertainties introduced from averaging included in the error bars.

**FIGURE 6:**
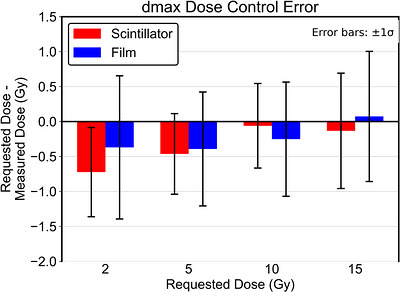
Control error based on deviation to requested dose entered into the BPF detector system for the scintillation detection system reading and film reading at 1.2 cm depth in solid water. For each requested dose, requested dose—measured dose are displayed as the average values over multiple trials, with the uncertainties introduced from averaging included in the error bars.

### 3.2 Scintillation cross calibration in FLASH

Scintillator cross calibration showed a linear relationship between measured charge and film dose, with calibration factors dependent on detector placement (Figure 4). Determined calibration factors were 5.33± 0.11 cGy/nC with BPF on the surface, and 3.67± 0.08 cGy/nC at depth. The ACR correction factor was determined to be 1.00± 0.015.

### 3.3 Pulse‐based control

For pulse‐based control, in comparing the two independent detection channels of the BPF detector, no discrepancy between the observed number of pulses measured was noted. The actual pulses measured, including those after the halting signal was sent, versus the requested pulses were in perfect agreement in 68% of tests (17 out of 25). A pulse error of +1 was observed in 20% of tests (5 out of 25), and a +2‐3‐pulse error was recorded in 12% of tests (3 out of 25). The pulse in which the MCU printed to have sent the halting signal agreed with the requested number of pulses in 100% of tests. Since the requested and MCU signal sent numbers matched perfectly, a conclusion can be made that pulse error is caused by hardware lag of the custom or LINAC circuitry, not the calculation speed of the MCU. Since the discrepancy in the number of pulses was 0–3, it can be concluded that the latency of the MLCHOLDOFF signal must be between 0 and 11 ms (i.e., 3 pulses / 360 Hz PRF + 2.7 ms (to account for the time right up until a fourth extra pulse, that is, 1 pulse /360 Hz rounded down to nearest tenth).

### 3.4 Dose‐based control

Dose‐based control demonstrated agreement with film measurements within 1.11±0.81 Gy and −1.45 ±0.38 Gy for surface and depth control, respectively (Figures 5 & 6). Surface‐based control exhibited slightly larger deviations at higher doses, while depth‐based control showed larger deviations at lower doses. Given the magnitude of uncertainties and limited sample size, these trends were not interpreted as statistically significant.

### 3.5 Dose‐per‐pulse dependence

**FIGURE 7:**
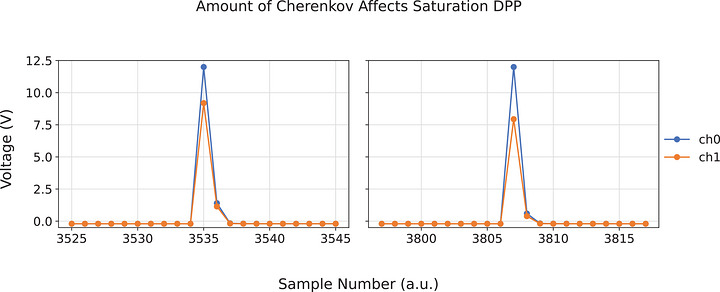
Raw data example illustrating different amounts of Cherenkov in ch1, but both signals are saturated.

The BPF system response was evaluated for DPP values (as reported by flashDiamond measurements) 0.015–0.78 Gy/p, as this was the range producible by the converted LINAC. A slightly larger dynamic range of BPF was observed for the 5 × 5 cm^2^ field compared to the 7 × 7 cm^2^ field. These results indicate that smaller field sizes may extend the usable dose‐per‐pulse range prior to saturation, since Cherenkov signal takes up less of the capacitor storage (Figure 7).

**FIGURE 8:**
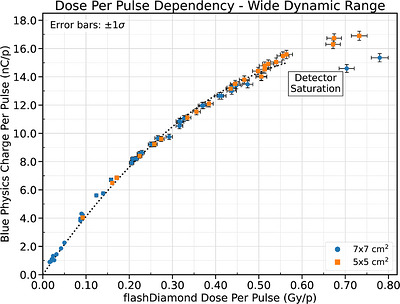
QPP versus DPP. Best quadratic fit: *y* = −31.525x^2^ + 44.349x.

The response dependence on DPP, shown in Figure 8, shows that there is a nonlinear response of the detector with DPP, best described with a quadratic fit (*R* ^2^ = 0.996). For this data, the fit was constrained to go through (0,0) to reflect the physical truth that the response is zero at zero DPP. A linear fit (not plotted, *y* = 30.66x) constrained to include a (0,0) point results in an *R* ^2^ of 0.936.

**FIGURE 9:**
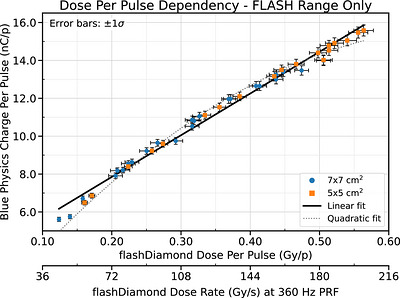
QPP versus DPP—FLASH range only. Linear fit: *y* = 22.021x + 3.444, Best quadratic fit: *y* = −30.529x^2^ + 43.888x.

Since our PRF is ∼360 Hz on average, this corresponds to a wide dynamic range of average dose rates 5.4–280.8 Gy/s. If the data is restricted to the pre‐saturation (of the detector) FLASH range (> 40 Gy/s, i.e., 0.11 Gy/p at 360 Hz), the detector response is well described by a linear model (*R* ^2^ = 0.9887), with only marginal improvement observed for a quadratic fit (*R* ^2^ = 0.9903): Δ*R* ^2^ = 0.0016 (Figure 9). This suggests that a linear approximation is sufficient for practical use within this range with small additional uncertainty. These fits were not constrained to a (0,0) point, to better model the actual data measured in this range. Due to the large baseline offset in the linear fit, there seems to be a change in the detector response between FLASH and CONV modes causing a quadratic approach between the dose rates in the wide dynamic range.

## 4 DISCUSSION

This work demonstrates the feasibility of using a modified scintillation detector as a real‐time beam termination mechanism for electron FLASH radiotherapy. The system is intended as an additional safety layer rather than a primary control system, addressing a critical gap of redundancy in FLASH control frameworks. By enabling in vivo, dose‐based termination, the BPF system is intended to mitigate the risk of gross delivery errors resulting from LINAC primary control system failure. The observed dose‐based control accuracy, with deviations within 1.45±0.38 Gy, supports this role as a redundant safety mechanism under the experimental conditions evaluated in this study.

To place this magnitude of error in a regulatory safety context, therapeutic radiation machine delivery errors are commonly reportable in U.S. state regulations as misadministration, medical events, or reportable events, depending on the jurisdiction. Under our state's therapeutic radiation machine regulations, reportable misadministration includes delivery to the wrong patient, wrong treatment modality, or wrong treatment site, as well as dose deviations in which the calculated weekly administered dose differs from the weekly prescribed dose by more than 30%, or the calculated total administered dose differs from the total prescribed dose by more than 20%.^17^ The registrant is required to notify the agency after discovery of misadministration. Using these criteria only as a representative regulatory benchmark, the measured dose‐control deviations in this study would fall below the 20% total‐dose reporting threshold for prescriptions greater than approximately 7.25 Gy, based on the largest observed deviation of 1.45 Gy, with similar implications for fractionated regimens. However, this comparison is intended only to contextualize the magnitude of the observed error relative to established reporting thresholds. It should not be interpreted as evidence that the BPF system is broadly generalizable or sufficient for primary control. Rather, the results support the potential role of the system as an independent, in vivo safety layer for reducing the likelihood of gross delivery errors in experimental and translational electron FLASH studies.

In addition to parameters presented in results, we also recorded the LINAC's water‐cooling system temperature over all readings, to monitor how the output changes with loading FLASH or environmental drifts. Our findings underscore the necessity of dose‐based control for redundant monitoring, demonstrating that pulse‐based control alone will be insufficient. On our LINAC system, a change in water cooling temperature reading of less than one‐degree Celsius caused by random fluctuation altered the LINAC output by greater than roughly 50 Gy/s. Therefore, if pulse‐based backup control were employed at 300 Hz with a request of 15 pulses, the actual dose delivered could vary between 5 (100 Gy/s/300 Hz * 15 pulses) and 7.5 Gy (150 Gy/s/300 Hz * 15 pulses) simply due to vault conditions at the time of delivery. Although water temperature is a crude surrogate for output variation, this result highlights that pulse‐based control does not deterministically map to delivered dose under FLASH conditions where DPP is unstable. In contrast, dose‐based control inherently compensates for such variations, reinforcing its necessity for independent safety systems.

Two primary limitations regarding stabilized DPP should be noted. First, dose delivery is inherently quantized to integer multiples of the DPP when operating under stable pulse conditions. As a result, even with perfect control, the delivered dose cannot be more precise than the final pulse increment. Future work will focus on enabling real‐time modulation of the final pulse through additional LINAC interfacing, allowing finer dose control. However, reducing the final pulse magnitude may result in instantaneous dose rates falling below the FLASH threshold, the implications of which remain unclear. Second, the system was only evaluated under conditions of stabilized DPP. While this reflects the intended operating regime for FLASH delivery, performance under unstable ramp‐up conditions was not assessed. Incorporating dynamic DPP tracking or correction into the control algorithm represents an important area for future development.

Other studies have investigated similar detectors for the primary control of FLASH beams. Ashraf et al.,^29^ evaluated the use of an Exradin W1 scintillation detector coupled to a custom field‐programmable gate array (FPGA) controller for both dose‐based and pulse‐based control. The controller would calculate DPP from the integrated W1 signal and subsequently terminate the LINAC beam delivery. Overall, the largest average control error reported between film and the scintillation‐based system was 1.40±0.40 Gy, which is comparable in magnitude to the dose‐control error observed in the present study of −1.45 ±0.38 Gy in similar conditions. In the Ashraf et al^29^ study, the dose‐based control inaccuracy appears to increase with total desired dose. This phenomenon was not observed clearly in the current study. Another key distinction of the current work is the use of an existing FDA‐approved detection system with minimal modifications to enable LINAC control, rather than reliance on a custom‐built controller that is difficult to reproduce or deploy broadly. Additionally, we propose a different role for scintillation‐based control, emphasizing its application as a redundant safety mechanism rather than as a primary control system, due to the inherently point‐like nature of such detectors. The comparison is intended only to contextualize the magnitude of the observed control error; it does not imply that the present or previously reported point‐detector system is appropriate for primary beam monitoring. Schneider et al.^31^ employed the standard commercially available Blue Physics scintillation detector with a similar connector circuit for beam control on an Elekta LINAC; However, in that study, control was limited to pulse‐based termination, most likely since dose‐based measurements were inaccurate without the modifications introduced in this study. Furthermore, pulse control accuracy was never explicitly quantified. In contrast, the present study extends this approach to dose‐based control by modifying the Blue Physics system to BPF. The present work extends this approach to a modified Varian LINAC, suggesting potential adaptability across vendor platforms, although further validation would be required. This study explicitly provides a direct evaluation of control accuracy for both pulse‐based and dose‐based methods using BPF.

The detector response exhibited a nonlinear dependence on DPP, which was not explicitly accounted for in the dose‐based control calculations. Although assuming linearity introduced only modest additional uncertainty in this study, incorporating this dependence into real‐time calculations may further improve accuracy. Given the demonstrated computational capacity of the MCU, implementation of a nonlinear correction model is feasible. However, this approach is constrained by detector saturation at higher signal levels. Increasing capacitor size could extend the dynamic range but would reduce temporal resolution, indicating a trade‐off that warrants further investigation.

An additional factor influencing detector performance is Cherenkov signal contribution. The results indicate that reducing Cherenkov generation increases the effective dynamic range of measurable DPP, as total charge accumulation in the capacitor determines saturation. Since Cherenkov signal occupies part of this dynamic range, reducing it allows more headroom for scintillation signal. In this study, measurements were performed at the field center to ensure stable dosimetry. In practice, in vivo placement near the field edge would reduce Cherenkov contribution and may further improve performance. Future work should quantify detector response under such conditions.

The BPF system was also observed to exhibit afterglow, characterized by a persistent signal above the noise floor following irradiation. This effect arises from longer‐lived excited states within the scintillator material.^48^ In this study, a threshold of −0.05 V was applied to exclude afterglow contributions, resulting in reproducible and dose‐proportional signals. The extent to which afterglow should be incorporated into dose calculations remains an open question and warrants further investigation.

In addition to afterglow, the BPF response was observed to vary with cumulative delivered dose. A separate preliminary investigation was conducted to assess the radiation hardness of the commercially available Blue Physics system. Large FLASH deliveries using time‐based control were administered to a brand‐new Blue Physics system. The delivered FLASH dose was estimated by multiplying the average DPP, measured using film prior to the study, by the number of pulses recorded by the scintillation acquisition unit. In between FLASH irradiations, the probe was irradiated under CONV reference conditions on a TrueBeam LINAC to monitor changes in detector response to a known dose as cumulative FLASH exposure increased. Overall, the study revealed an estimated average signal degradation of 1.9 %/kGy evaluated up to 30 kGy. In parallel, both the ACR correction factor and the signal from the Cherenkov‐only channel were monitored. The ACR factor remained within 5.3%. In contrast, the Cherenkov signal exhibited an average 0.66%/kGy reduction, supporting the prevailing hypothesis that radiation‐induced yellowing of the optical fiber attenuates the transmitted light signal, while the fiber itself remains structurally intact relative to the scintillator.^28^ This degradation value was not included as a separate component in the uncertainty budget because the radiation hardness assessment was preliminary and is intended primarily to disclose a potential limitation of the system rather than to provide a rigorously validated uncertainty model. During the measurements used for dose quantification in this study, the BPF system received ∼400 Gy, corresponding to a worst‐case signal change of approximately 0.76% based on the observed degradation rate. Given the magnitude of the other uncertainty components, this contribution was considered negligible for the present dataset.

Radiation‐induced scintillator degradation and its recovery in the short‐ and long‐term time periods are complex effects and likely depend on multiple interacting factors, including accumulated dose, dose rate, detector composition, irradiation history, and rest period between irradiations. Although beam quality may contribute to this behavior, the available literature does not suggest a simple dependence on beam quality alone. For example, Giguere et al.^49^ evaluated plastic scintillation dosimeters exposed to 200 MeV UHDR electrons and reported light‐output decreases of <1.87%/kGy after cumulative 37.2 kGy irradiation, along with short term recovery that depended on the rest duration and accumulated dose and partial long‐term recovery after extended rest. This degradation is comparable to the 1.9%/kGy observed in the present lower‐energy electron FLASH study, suggesting that factors other than beam energy alone may strongly influence the measured sensitivity loss. Our preliminary determined degradation value is less than or comparable to other radiation hardness studies on other scintillation models as well. One study reports that the Exradin W2 scintillator decays 2%/kGy claiming this is a “minor inherent limitation,” citing the manufacturer manual as reference.^30^ However, this manual most likely evaluated decay in lower dose rates. Ashraf et al.^29^ found a 16.2%/kGy loss in the first kGy of irradiation to the previous Exradin W1 model in FLASH dose rates. In future experiments, they observed a roughly ∼ 7%/kGy degradation, and even some recovery between separate data collections. Lastly, a study examined the radiation degradation of the HYPERSCINT RP‐FLASH scintillator, finding a decay of 20% after 10kGy, roughly 2%/kGy on average.^50^ Similarly to the conclusions made by other radiation hardness scintillation research discussed above, the observed radiation‐induced degradation indicates that, in its current form, frequent recalibration of BPF against film under FLASH conditions would be required to maintain accurate dose measurements. Frequent recalibration would increase workload of users and impact on the uncertainty. Therefore, the degradation measurements in this work should be interpreted as preliminary and included for transparency. Further work will require systematic characterization of degradation and recovery as functions of accumulated dose, dose rate, rest period, beam conditions, and detector composition. Our primary next objective is to identify a scintillation compound that does not exhibit afterglow effects in FLASH or signal degradation over a practical range of cumulative dose. We also are experimenting with changes to the architecture of the device to combat this signal decay.

In this study, we chose to minimally modify an existing commercial scintillation detector to be compatible with FLASH and easily hook up the detector to the LINAC to improve reproducibility and translational ability instead of starting from the ground up. Therefore, we opted to use the on board MCU in the Blue Physics system, opposed to a field programmable gate array (FPGA) based controller. However, our study showed that the MCU was temporally sufficient to measure pulses, calculate dose, and send a halting signal before the next pulse was measured in 100% of tests. Therefore, it does not appear that the controller was causing the latency. In fact, FPGA‐based upgrades may not improve our system's inaccuracy. In a thesis,^51^ while high accuracy was shown on the Mobetron platform at a relatively low 90 Hz PRF, the Trilogy experimentations with higher PRF explicitly reports “frequent overshoots where additional, unwanted pulses were produced due to latencies in the beam‐hold systems.” This indicates that termination overshoot was governed by LINAC gating latency and not resolved with FPGA‐based processing. In another study,^29^ a –1 pulse inaccuracy was observed at repetition frequencies comparable to those used here, and 100% pulse agreement was achieved only at much lower PRFs. Additionally, dose‐control accuracy reported was of similar magnitude to our findings as mentioned. The LINAC PRF during their dose‐based control testing was not specified, limiting direct comparison. Lempart et al.^51^ used a MCU from Atmega to count pulses from a diode detector. They achieved perfect pulse accuracy. This was because they chose the LINAC termination carefully, stopping the trigger pulse from reaching the thyratron with a custom circuit which showed extremely small latency.

We decided to use MLCHOLDOFF since this signal is available on all Varian LINACs and was very straightforward for future users to mimic. However, the latency from this signal was majorly causing the discrepancies between requested and obtained dose. Although we did not directly measure the latency from MLCHOLDOFF, we can estimate that it could sometimes be on the order of 11 ms to cause an additional 3 pulses to be let out. In the future, we plan to no longer use this pathway due to latency observations regardless. Instead, we plan to recommission respiratory gating‐based control for even better translation ability since this is already a vendor accepted pathway for external collaboration with LINAC systems. It is unclear if this will be faster. Another method would be to mimic the Lempart et al.^52^ technique. However, this involved custom circuitry which is not easily replicable, and is more “invasive” to the LINAC which may cause vendor‐pushback in the future. More importantly, the study evaluates control on an Elekta LINAC. It is unclear if a similar circuit could be developed on Varian platforms.

While this work is primarily focused on FLASH beamline safety mechanisms, the proposed systems have broader applicability. Most directly, the BPF detector will be a valuable tool for FLASH QA. Beyond FLASH applications, the concept of an active in vivo dose monitor may have further relevance for CONV LINAC workflows, such as total body and total skin electron irradiation (TBI/TSE). While CONV LINACs have timer‐based backup termination that is more than sufficient, it does not directly determine the dose delivered to the patient but only helps for estimation and catastrophic overdose. Delivered dose in these modalities is often associated with relatively large uncertainty, and dose verification is typically performed retrospectively using thermoluminescence or optically stimulated luminescence detectors.^52^ In contrast, the proposed active probe enables real‐time monitoring and can terminate treatment when a deviation from the intended dose is detected. Additionally, unlike many previously investigated faster responding in vivo detectors for TBI/TSE, this system does not require detector bias, thereby offering an additional safety advantage.^53^ The system would not serve to replace backup timer‐based control systems but instead add another layer of redundancy that directly monitors delivered dose and terminates the beam for increased patient safety and dose accuracy. In essence, we introduce a new category of in vivo dosimetry: “Active QA.”

## 5 CONCLUSIONS

A modified plastic scintillation detector system was implemented as a real‐time in vivo beam termination mechanism for electron FLASH radiotherapy under stable DPP conditions on a single modified Varian LINAC. The system demonstrated pulse and dose‐based termination capability, with termination accuracy limited primarily by downstream hardware latency. While not intended for primary beam control, the system may provide an additional in vivo safety layer for mitigating gross delivery errors in experimental and translational FLASH studies. Further validation across beam conditions and platforms is required before broader clinical implementation can be considered.

## FUNDING INFORMATION

This work was part of “Radiation Therapy Monitoring” project supported by VCU Intellectual property foundation through the 2025 VCU commercialization fund.

## CONFLICT OF INTEREST STATEMENT

The authors declare no conflicts of interest.

## Data Availability

Authors will share data upon request to the corresponding author.

## APPENDIX A Film calibration curves

### A.1

To fit the mean red pixel values versus CONV dose of EBT3 and EBT‐XD sheets of film, the Rodbard function of the form in Equation A1 was used.

(A1)
$$D=c\cdot {\left(\frac{b-a}{\left(PV-a\right)\;-\;1}\right)}^{\left(1/d\right)},$$

where D is the dose in cGy, PV is the mean red pixel value, and *a*, *b*, *c*, *d* are fitting parameters. For EBT3 and EBT‐XD, fitting parameters were *a* = 8.773.2, *b* = 45080.1, *c* = 340.41, *d* = 0.93 with *R* ^2 ^= 0.999 and a fit uncertainty of 1.65%, and *a* = 5451.72, *b* = 46303.77, *c* = 976.64, *d* = 0.96, with *R* ^2 ^= 0.999 and a fit uncertainty of 1.89%, respectively.

## References

[1] Favaudon V , Caplier L , Monceau V , et al. Ultrahigh dose‐rate FLASH irradiation increases the differential response between normal and tumor tissue in mice. Sci Transl Med. 2014;6(245):245ra93. doi:10.1126/scitranslmed.3008973 25031268

[2] Hornsey S , Alper T . Unexpected dose‐rate effect in the killing of mice by radiation. Nature. 1966;210(5032):212‐213. doi:10.1038/210212a0 5962093

[3] Field SB , Bewley DK . Effects of dose‐rate on the radiation response of rat skin. Int J Radiat Biol Relat Stud Phys Chem Med. 1974;26(3):259‐267. doi:10.1080/09553007414551221 4547756

[4] Hageman E , Che PP , Dahele M , Slotman BJ , Sminia P . Radiobiological aspects of FLASH Radiotherapy. Biomolecules. 2022;12(10):1376. doi:10.3390/biom12101376 36291585 PMC9599153

[5] Montay‐Gruel P , Petersson K , Jaccard M , et al. Irradiation in a flash: unique sparing of memory in mice after whole brain irradiation with dose rates above 100 Gy/s. Radiother Oncol. 2017;124(3):365‐369. doi:10.1016/j.radonc.2017.05.003 28545957

[6] Vozenin MC , Hendry JH , Limoli CL . Biological benefits of ultra‐high dose rate FLASH radiotherapy: sleeping beauty awoken. Clin Oncol. 2019;31(7):407‐415. doi:10.1016/j.clon.2019.04.001 PMC6850216 31010708

[7] Levy K , Natarajan S , Wang J , et al. Abdominal FLASH irradiation reduces radiation‐induced gastrointestinal toxicity for the treatment of ovarian cancer in mice. Sci Rep. 2020;10(1):21600. doi:10.1038/s41598-020-78017-7 33303827 PMC7728763

[8] Smyth LML , Donoghue JF , Ventura JA , et al. Comparative toxicity of synchrotron and conventional radiation therapy based on total and partial body irradiation in a murine model. Sci Rep. 2018;8(1):12044. doi:10.1038/s41598-018-30543-1 30104646 PMC6089899

[9] Beyreuther E , Brand M , Hans S , et al. Feasibility of proton FLASH effect tested by zebrafish embryo irradiation. Radiotherapy and Oncology. 2019;139:46‐50. doi:10.1016/j.radonc.2019.06.024 31266652

[10] Venkatesulu BP , Sharma A , Pollard‐Larkin JM , et al. Ultra high dose rate (35 Gy/sec) radiation does not spare the normal tissue in cardiac and splenic models of lymphopenia and gastrointestinal syndrome. Sci Rep. 2019;9(1):17180. doi:10.1038/s41598-019-53562-y 31748640 PMC6868225

[11] Friedl AA , Prise KM , Butterworth KT , Montay‐Gruel P , Favaudon V . Radiobiology of the FLASH effect. Med Phys. 2022;49(3):1993‐2013. doi:10.1002/mp.15184 34426981

[12] Favaudon V , Labarbe R , Limoli CL . Model studies of the role of oxygen in the FLASH effect. Med Phys. 2022;49(3):2068‐2081. doi:10.1002/mp.15129 34407219 PMC8854455

[13] Ashraf MR , Rahman M , Zhang R , et al. Dosimetry for FLASH radiotherapy: a review of tools and the role of radioluminescence and Cherenkov emission. Front Phys. 2020;8:328. doi:10.3389/fphy.2020.00328

[14] Romano F , Bailat C , Jorge PG , Lerch MLF , Darafsheh A . Ultra‐high dose rate dosimetry: challenges and opportunities for FLASH radiation therapy. Med Phys. 2022;49(7):4912‐4932. doi:10.1002/mp.15649 35404484 PMC9544810

[15] Siddique S , Ruda HE , Chow JCL . FLASH radiotherapy and the use of radiation dosimeters. Cancers. 2023;15(15):3883. doi:10.3390/cancers15153883 37568699 PMC10417829

[16] DeFrancisco J , Kim S . A systematic review of electron FLASH dosimetry and beam control mechanisms utilized with modified non‐clinical LINACs. J Applied Clin Med Phys. 2025;26(4):e70051. doi:10.1002/acm2.70051 40108673 PMC11969112

[17] Virginia, Department of Health . 12VAC5‐481‐3430. Therapeutic Radiation Machines—Photon Therapy Systems (500 kV and Above) and Electron Therapy Systems (500 kV and Above) . Virginia Administrative Code, 2024. Accessed 15 Jan. 2026 law.lis.virginia.gov/admincode/title12/agency5/chapter481/section3430/

[18] Cetnar AJ , DiCostanzo DJ . The lifetime of a linac monitor unit ion chamber. J Applied Clin Med Phys. 2021;22(12):108‐114. doi:10.1002/acm2.13463 PMC8664141 34762336

[19] Medical Electrical Equipment—Part 2: Particular Requirements for the Basic Safety and Essential Performance of High Frequency Surgical Equipment and High Frequency Surgical Accessories . IEC 60601–2:2009, International Electrotechnical Commission, 2009.

[20] Konradsson E , Ceberg C , Lempart M , et al. Correction for ion recombination in a built‐in monitor chamber of a clinical linear accelerator at ultra‐high dose rates. Radiat Res. 2020;194(6):580‐586. doi:10.1667/RADE-19-00012 33348371 PMC7612000

[21] Szpala S , Huang V , Zhao Y , et al. Dosimetry with a clinical linac adapted to FLASH electron beams. J Applied Clin Med Phys. 2021;22(6):50‐59. doi:10.1002/acm2.13270 34028969 PMC8200504

[22] Liu K , Holmes S , Hooten B , Schüler E , Beddar S . Evaluation of ion chamber response for applications in electron FLASH radiotherapy. Med Phys. 2024;51(1):494‐508. doi:10.1002/mp.16726 37696271 PMC10840726

[23] Shelton J , Kumar GP . Comparison between auditory and visual simple reaction times. NM. 2010;01(01):30‐32. doi:10.4236/nm.2010.11004

[24] Konradsson E , Wahlqvist P , Thoft A , et al. Beam control system and output fine‐tuning for safe and precise delivery of FLASH radiotherapy at a clinical linear accelerator. Front Oncol. 2024;14:1342488. doi:10.3389/fonc.2024.1342488 38304871 PMC10830783

[25] Rahman M , Ashraf MR , Zhang R , et al. Electron FLASH delivery at treatment room isocenter for efficient reversible conversion of a clinical LINAC. Int J Radiat Oncol Biol Phys. 2021;110(3):872‐882. doi:10.1016/j.ijrobp.2021.01.011 33444695 PMC10416223

[26] Sloop A , Ashraf MR , Rahman M , et al. Rapid switching of a C‐series linear accelerator between conventional and ultrahigh‐dose‐rate research mode with beamline modifications and output stabilization. Int J Radiat Oncol Biol Phys. 2024;119(4):1317‐1325. doi:10.1016/j.ijrobp.2024.01.215 38552990 PMC12356227

[27] Bernelin T , Muir B , Renaud J , et al. Characterization of a shielded beam current transformer for ultra‐high dose rate (FLASH) electron beam monitoring and dose reporting. Medical Physics. 2025;52(7):e17927. doi:10.1002/mp.17927 40473411 PMC12257996

[28] Liu K , Palmiero A , Chopra N , et al. Dual beam‐current transformer design for monitoring and reporting of electron ultra‐high dose rate (FLASH) beam parameters. J Applied Clin Med Phys. 2023;24(2):e13891. doi:10.1002/acm2.13891 36601691 PMC9924113

[29] Ashraf MR , Rahman M , Cao Xu , et al. Individual pulse monitoring and dose control system for pre‐clinical implementation of FLASH‐RT. Phys Med Biol. 2022;67(9):095003. doi:10.1088/1361-6560/ac5f6f PMC10305796 35313290

[30] Oh K , Hyun MA , Gallagher KJ , Yan Y , Zhou S . Characterization of a commercial plastic scintillator for electron FLASH dosimetry. J Applied Clin Med Phys. 2024;25(8):e14451. doi:10.1002/acm2.14451 38952057 PMC11302813

[31] Schneider F , Bauer CJ , Göbel ID , et al. Rapid and reversible adaptation of a clinical linear accelerator for electron FLASH radiotherapy. Physica Medica. 2025;136:105032. doi:10.1016/j.ejmp.2025.105032 40554908

[32] DeFrancisco J . Establishing a Conventional LINAC‐Based Electron FLASH Beam. VCU Theses and Dissertations. 7937. Published online 2025. doi:10.25772/Q8ST-C928

[33] Niroomand‐Rad A , Chiu‐Tsao S‐T , Grams MP , et al. Report of AAPM Task Group 235 radiochromic film dosimetry: an update to TG‐55. Med Phys. 2020;47(12):5986‐6025. doi:10.1002/mp.14497 32990328

[34] Guan F , Wang X , Yang M , et al. Dosimetric response of Gafchromic™ EBT‐XD film to therapeutic protons. Precis Radiat Oncol. 2023;7(1):15‐26. doi:10.1002/pro6.1187 37868341 PMC10586355

[35] Howard ME , Herman MG , Grams MP . Methodology for radiochromic film analysis using FilmQA Pro and ImageJ. PLoS ONE. 2020;15(5):e0233562. doi:10.1371/journal.pone.0233562 32437474 PMC7241712

[36] León‐Marroquín EY , Mulrow D , Darafsheh A , Khan R . Response characterization of EBT‐XD radiochromic films in megavoltage photon and electron beams. Med Phys. 2019;46(9):4246‐4256. doi:10.1002/mp.13708 31297824

[37] Al Khalifa M , Ma T , Aljuaid H , Kim S , Song WY . A novel Cherenkov radiation removal method for plastic scintillator detectors in a 0.35 T MR‐Linac. J Applied Clin Med Phys. 2025;26(8):e70202. doi:10.1002/acm2.70202 40817237 PMC12356693

[38] Marinelli M , Felici G , Galante F , et al. Design, realization, and characterization of a novel diamond detector prototype for FLASH radiotherapy dosimetry. Med Phys. 2022;49(3):1902‐1910. doi:10.1002/mp.15473 35064594 PMC9306529

[39] Farrance I , Frenkel R . Uncertainty of measurement: a review of the rules for calculating uncertainty components through functional relationships. Clin Biochem Rev. 2012;33(2):49‐75. 22896744 PMC3387884

[40] Jaccard M , Petersson K , Buchillier T , et al. High dose‐per‐pulse electron beam dosimetry: usability and dose‐rate independence of EBT3 Gafchromic films. Med Phys. 2017;44(2):725‐735. doi:10.1002/mp.12066 28019660

[41] Del Sarto D , Masturzo L , Cavalieri A , et al. A systematic investigation on the response of EBT‐XD gafchromic films to varying dose‐per‐pulse, average dose‐rate and instantaneous dose‐rate in electron flash beams. Front Phys. 2025;13:1474416. doi:10.3389/fphy.2025.1474416

[42] Saw CB , Pawlicki T , Korb LJ , Wu A . Effects of extended SSD on electron‐beam depth‐dose curves. Med Dosim. 1994;19(2):77‐81. doi:10.1016/0958-3947(94)90075-2 7916979

[43] Arunkumar T , Supe S , Ravikumar M , Sathiyan S , Ganesh M . Electron beam characteristics at extended source‐to‐surface distances for irregular cut‐outs. J Med Phys. 2010;35(4):207. doi:10.4103/0971-6203.71763 21170185 PMC2990115

[44] Shameem T , Bennie N , Butson M , Thwaites D . Comparative characterisation of different types of Gafchromic films for radiotherapy use. Phys Eng Sci Med. 2025;48(3):1425‐1437. doi:10.1007/s13246-025-01596-0 40658329 PMC12511257

[45] Alnawaf H , Yu PKN , Butson M . Comparison of Epson scanner quality for radiochromic film evaluation. J Applied Clin Med Phys. 2012;13(5):314‐321. doi:10.1120/jacmp.v13i5.3957 PMC5718226 22955661

[46] Ferreira BC , Lopes MC , Capela M . Evaluation of an Epson flatbed scanner to read Gafchromic EBT films for radiation dosimetry. Phys Med Biol. 2009;54(4):1073‐1085. doi:10.1088/0031-9155/54/4/017 19168937

[47] Marroquin EYL , Herrera González JA , Camacho López MA , Barajas JEV , García‐Garduño OA . Evaluation of the uncertainty in an EBT3 film dosimetry system utilizing net optical density. J Applied Clin Med Phys. 2016;17(5):466‐481. doi:10.1120/jacmp.v17i5.6262 27685125 PMC5874103

[48] Viererbl L , Kolros A , Vratislavská HA . Long‐term afterglow of solid scintillators. Radiat Prot Dosimetry. 2022;198(9‐11):666‐669. doi:10.1093/rpd/ncac116 36005988

[49] Giguère C , Hart A , Bateman J , et al. Radiation damage and recovery of plastic scintillators under ultra‐high dose rate 200 MeV electrons at CERN CLEAR facility. Phys Med Biol. 2025;70(7):075012. doi:10.1088/1361-6560/adc234 40101360

[50] Guo L , Zhou B , Tsai Y , Jiang K , Iakovenko V , Wang KK . Comprehensive characterization and validation of a fast‐resolving (1000 Hz) plastic scintillator for ultra‐high dose rate electron dosimetry. Med Phys. 2025;52(10):e70006. doi:10.1002/mp.70006 40983874 PMC12454735

[51] Sloop, AM , Developing Real‐time, Online Beam Control of UHDR Irradiators to Facilitate FLASH Translational Investigations . Dissertation. Dartmouth College; 2025. 434. https://digitalcommons.dartmouth.edu/dissertations/434

[52] Lempart M , Blad B , Adrian G , et al. Modifying a clinical linear accelerator for delivery of ultra‐high dose rate irradiation. Radiother Oncol. 2019;139:40‐45. doi:10.1016/j.radonc.2019.01.031 30755324

[53] Dyk JV , Galvin JM , Glasgow GP , Podgorsak EB . The Physical Aspects of Total and Half Body Photon Irradiation. AAPM; 1986. doi:10.37206/16

